# Herpesviridae and Atypical Bacteria Co-Detections in Lower Respiratory Tract Samples of SARS-CoV-2-Positive Patients Admitted to an Intensive Care Unit

**DOI:** 10.3390/microorganisms12040714

**Published:** 2024-03-31

**Authors:** Gašper Grubelnik, Miša Korva, Rok Kogoj, Tina Polanc, Matej Mavrič, Monika Jevšnik Virant, Tina Uršič, Darja Keše, Katja Seme, Miroslav Petrovec, Matjaž Jereb, Tatjana Avšič-Županc

**Affiliations:** 1 Institute of Microbiology and Immunology, Faculty of Medicine, University of Ljubljana, Zaloška Cesta 4, 1000 Ljubljana, Slovenia; gasper.grubelnik@mf.uni-lj.si (G.G.); misa.korva@mf.uni-lj.si (M.K.); rok.kogoj@mf.uni-lj.si (R.K.); monika.jevsnik@mf.uni-lj.si (M.J.V.); tina.ursic@mf.uni-lj.si (T.U.); darja.kese@mf.uni-lj.si (D.K.); katja.seme@mf.uni-lj.si (K.S.); mirc.petrovec@mf.uni-lj.si (M.P.); 2 Department of Infectious Diseases, Ljubljana University Medical Center, Japljeva Ulica 2, 1000 Ljubljana, Slovenia; matej.mavric@kclj.si (M.M.); matjaz.jereb@kclj.si (M.J.); 3 Faculty of Medicine, University of Ljubljana, Vrazov Trg 2, 1000 Ljubljana, Slovenia

**Keywords:** SARS-CoV-2, co-detections, coinfections, lower respiratory tract, ICU, herpesviruses, atypical bacteria

## Abstract

Shortly after the emergence of severe acute respiratory syndrome coronavirus 2 (SARS-CoV-2), cases of viral, bacterial, and fungal coinfections in hospitalized patients became evident. This retrospective study investigates the prevalence of multiple pathogen co-detections in 1472 lower respiratory tract (LRT) samples from 229 SARS-CoV-2-positive patients treated in the largest intensive care unit (ICU) in Slovenia. In addition to SARS-CoV-2, (rt)RT-PCR tests were used to detect cytomegalovirus (CMV), Epstein–Barr virus (EBV), herpes simplex virus 1 (HSV-1), herpes simplex virus 2 (HSV-2), varicella zoster virus (VZV), and atypical bacteria: *Chlamydia pneumoniae*, *Mycoplasma pneumoniae* and *Legionella pneumophila/*spp. At least one co-detection was observed in 89.1% of patients. EBV, HSV-1, and CMV were the most common, with 74.7%, 58.1%, and 38.0% of positive patients, respectively. The median detection time of EBV, HSV-1, and CMV after initial SARS-CoV-2 confirmation was 11 to 20 days. Bronchoalveolar lavage (BAL) and tracheal aspirate (TA) samples showed equivalent performance for the detection of EBV, CMV, and HSV-1 in patients with both available samples. Our results indicate that SARS-CoV-2 infection could be a risk factor for latent herpesvirus reactivation, especially HSV-1, EBV, and CMV. However, additional studies are needed to elucidate the clinical importance of these findings.

## 1. Introduction

Soon after the onset of severe acute respiratory syndrome coronavirus 2 (SARS-CoV-2) in Wuhan, China [1], the first patients were being hospitalized and coinfections became evident and an important research topic, especially in critically ill patients in intensive care units (ICU) [1,2]. The treatment of such patients is not only invasive but often requires immunomodulatory agents that can induce an altered immune system response, leading to a higher risk of additional infections and reactivations of latent viral infections [3,4,5].

In this context, some members of the *Herpesviridae* family—herpes simplex virus 1/2 (HSV-1/2), varicella zoster virus (VZV), cytomegalovirus (CMV), and Epstein–Barr virus (EBV)—come to attention due to their high prevalence in the general population and reactivation capabilities [4,6]. Herpesviruses cause chronic infections because a latent infection is established after the primary lytic infection. Various stimuli, such as immunosuppressive treatment, the damage caused by disease, microtrauma caused by endotracheal tubes, and so on, can cause reactivation of herpesviruses and, therefore, infectious virus production. After SARS-CoV-2 infection, various hyperinflammatory events occur with immune system dysregulation, which may serve as a potential gateway for latent herpesvirus reactivation [4,5,6]. Not only may herpesvirus reactivations contribute to the severity of SARS-CoV-2 infection [6], but ICU SARS-CoV-2 patients with herpesvirus reactivation are also more vulnerable to bacterial superinfections [4].

With regard to additionally acquired bacterial superinfections, the atypical bacteria *Chlamydia pneumoniae*, *Mycoplasma pneumoniae*, and *Legionella pneumophila/*spp. were of interest because of data from some previous studies [7,8,9,10,11]. *C. pneumoniae* and *M. pneumoniae* usually cause mild respiratory infections and, only occasionally, severe complications in some patients [8,9]. However, *C. pneumoniae*, *M. pneumoniae*, and *L. pneumophila/*spp. can cause atypical pneumonia, which may induce severe clinical manifestations. Despite the fact that atypical bacteria are not as frequently detected as viral superinfections, their importance should not be overlooked [7,9,12].

A number of previous studies on SARS CoV-2-positive patients and coinfections with CMV [5,13,14,15,16,17,18], EBV [15,16,19,20,21], and HSV-1 [5,13,17,18,22,23,24,25] are available. On the other hand, only a limited number of reports for HSV-2 [13,22] and VZV [18,26] are available. However, most of these studies were performed on upper respiratory samples or blood or are based on serology data. Studies of co-detections in lower respiratory tract samples (LRT) and longitudinal follow-up samples from an analytical point of view approached by a direct method of pathogen detection are scarce, but they are greatly needed to better estimate the impact and dynamics of viral and bacterial co-detections in COVID-19 patients.

Therefore, the aim of the study was to retrospectively determine co-detections of CMV, EBV, HSV-1/2, VZV, *C. pneumoniae*, *M. pneumoniae*, and *L. pneumophila/*spp. in LRT samples of severe COVID-19 patients and to determine the frequency and type of such co-detections. Additionally, time between the first SARS-CoV-2 detection and co-detection was also assessed. Furthermore, a comparison between TA and BAL samples from the same patient for consistency in specific herpesvirus detection was performed. Finally, we explored if SARS-CoV-2 infection could be one of the risk factors for latent herpesvirus reactivation in LRT samples of severely ill patients admitted to the ICU.

## 2. Materials and Methods

### 2.1. Selection of Patients and Samples

Between 1 March 2020 and 15 December 2021, a total of 665 COVID-19 patients were hospitalized in the ICU of the Department of Infectious Diseases at the Ljubljana University Medical Center, the largest tertiary hospital and main teaching hospital in Slovenia. All collected LRT samples were collected for SARS-CoV-2 diagnostic and follow-up purposes.

For the study, a subset of 229 patients (171 men, 58 women) with at least three consecutive LRT samples (between 3 to 18 samples per patient) were included. A total of 1187 tracheal aspirates (TA), 199 bronchoalveolar lavages (BAL), 29 bronchoaspirates (BA), and 57 sputum (SP) samples collected at different timepoints during their stay in the ICU were identified and selected for retrospective analysis on herpesviruses and atypical bacteria. The mean age of the included patients was 63.8 ± 10.4 years, with an age range from 28 to 84 years. More specifically, 11.8% (27/229) of patients were younger than 50 years, 54.1% (124/229) were between 50 and 69 years of age, and 34.1% (78/229) of patients were 70 or older. Clinical data on the disease outcome (survived/deceased), the application of immunosuppressive treatment, and/or antiviral treatment were also collected and included in the final analysis.

This study was performed in accordance with the ethical guidelines for human research, the World Medical Association’s Declaration of Helsinki, the Oviedo Convention on Human Rights and Biomedicine, and the Slovenian Code on Medical Deontology. The study was approved by the National Medical Ethics Committee, Ministry of Health, Republic of Slovenia (0120 211/2020/7).

### 2.2. Total Nucleic Acid Isolation and (rt)RT-PCR

The total nucleic acids were isolated using the MagNA Pure 96 DNA and Viral NA Small Volume Kit (Roche Applied Science, Mannheim, Germany) according to the manufacturer’s instructions. All subsequent (rt)RT-PCR reactions were performed with the same eluate of the respective sample.

Multiplex reverse-transcription real-time PCR (rtRT-PCR) was performed for the detection of SARS-CoV-2 using a Lightmix^®^ Kit SARS-CoV-2 E+N UBC (Roche Applied Science, Mannheim, Germany). Real-time PCR (RT-PCR) was performed for the detection of selected members of the Herpesviridae family using commercially available RT-PCR kits (GeneProof a. s., Brno, Czech Republic), and for *C. pneumoniae*, *M. pneumoniae*, and *L. pneumophila/*spp. detection, multiplex RT PCR was performed using LightCycler^®^ Multiplex DNA Master (Roche Applied Science, Mannheim, Germany) and LightMix^®^ Modular primers (TIB Molbiol, Berlin, Germany). Additional details of the procedures used are available in Appendix A.

### 2.3. Data Analysis

In the analysis, cycle threshold (Ct) values were used, which refer to the number of cycles in a PCR reaction needed to replicate enough PCR product to be detected (crosses a threshold line). Maximum Ct value spans were calculated for each positive patient from the corresponding sample with the lowest Ct value as an absolute Ct difference between the Ct value of the sample and Ct value of the limit of detection (Ct = 40). Maximum spans were arbitrarily divided into five groups by an increment of five Ct values to mitigate sample type and quality of collection difference (0–5.0, 5.1–10.0, 10.1–15.0, 15.1–20.0, 20.1–25.4). The normality of the data distributions was assessed with Q–Q plots and the Shapiro–Wilk test. The distribution of variables informed the decision on using parametric or non-parametric statistical tests. The differences in maximum Ct value spans between different patient groups (survived: deceased, immunosuppressive treatment: no immunosuppressive treatment, antiviral treatment: no antiviral treatment) were assessed with a pairwise Welch’s *t*-test. The differences in the number of co-detections between patient groups were assessed with a pairwise Wilcoxon rank sum test. Multiple comparison correction was performed using the false discovery rate (FDR).

The threshold for statistical significance was set at *p* < 0.05 in all cases. Data analyses were performed using Microsoft^®^ Excel^®^ 2016 version 16.0.5356.1000 (Microsoft Corporation, Redmond, WA, USA) and R statistical software version 4.3.1 (The R Foundation for Statistical Computing, Vienna, Austria).

## 3. Results

### 3.1. Sample Type Structure

The samples included were sorted according to sample type and the number of consecutive samples per patient for better insight into the sample type structure in the study. The majority of available samples were TA, available for 98.7% of patients. BAL samples were available for 48.9% of patients (24.9% of patients had one BAL sample, 16.2% two, 3.0% three, 4.4% four, and 0.4% seven). The SP and BA samples were available only for 17.5% and 10.0% of patients, respectively.

A stratification analysis of consecutive samples per patient shows that the majority of patients (93.5%) had 3 to 10 LRT samples available. Patients with a higher number of consecutive samples (maximum 18) were fewer (Table 1).

### 3.2. Clinical Data Analysis of SARS-CoV-2-Positive Patients

Among the 229 patients included, the mortality rate was 25.8%; clinical criteria are listed in Table A1—Appendix B. Due to severe disorder of the immune system, 22 (9.6%) patients received immunosuppressive treatment: rituximab and other biologics that act against B lymphocytes (anti-CD20, anti-CD38, anti-CD52, proteasome inhibitors, chimeric antigen receptor (CAR) T-cell cell therapy directed against antigens on the surface of B lymphocytes); antimetabolites, alkylating agents, methylprednisolone (or equivalent) ≥ 16 mg > 14 days. Antiviral treatment was administered to 34 (14.8%) patients. Detailed clinical data are presented in Table 2.

Serology data of herpesvirus infection were available for the majority of patients who were found to be HSV-1, EBV, or CMV positive in LRT samples included in this study. More accurately, for 125/133 (94.0%), 157/171 (91.8%), and 77/87 (88.5%) patients, serology data were available for HSV-1, EBV, and CMV, respectively. HSV-1 specific IgG antibodies were detected in 124 patients. One patient had no HSV-1 antibodies detected (neither IgG nor IgM). EBV specific antibodies (IgG EBNA/VCA) were detected in 155 patients. Two patients had no specific EBV antibodies detected (neither IgG EBNA/VCA/EA nor IgM). CMV specific IgG antibodies were detected in 75 patients. Two patients had no specific CMV antibodies detected (neither IgG nor IgM).

### 3.3. Overall Results of SARS-CoV-2, Herpesviruses, and Atypical Bacteria Detection in Lower Respiratory Tract Samples

SARS-CoV-2 infection was confirmed by rtRT-PCR in all 229 patients enrolled; however, since multiple consecutive follow-up samples were available for individual patients, in some samples, SARS-CoV-2 was not detected, 187/1472 (12.7%). The overall results of RT-PCR for the detection of selected herpesvirus family members showed that EBV, HSV-1, and CMV were detected in 171 (74.7%), 133 (58.1%), and 87 (38%) patients, respectively, with corresponding positive samples for EBV, HSV-1, and CMV: 821 (55.8%), 579 (39.3%), and 347 (23.6%), respectively. On the other hand, VZV and HSV-2 were detected in two patients; that is, nine (0.6%) and eight (0.5%) samples, respectively. *M. pneumoniae* and *L. pneumophila* were detected in one patient: in five (0.3%) and three (0.2%) samples, respectively. *C. pneumoniae* was not detected in any sample.

### 3.4. Ct Value Distribution Analysis of Detected Herpesviruses and Atypical Bacteria

**Figure 1:**
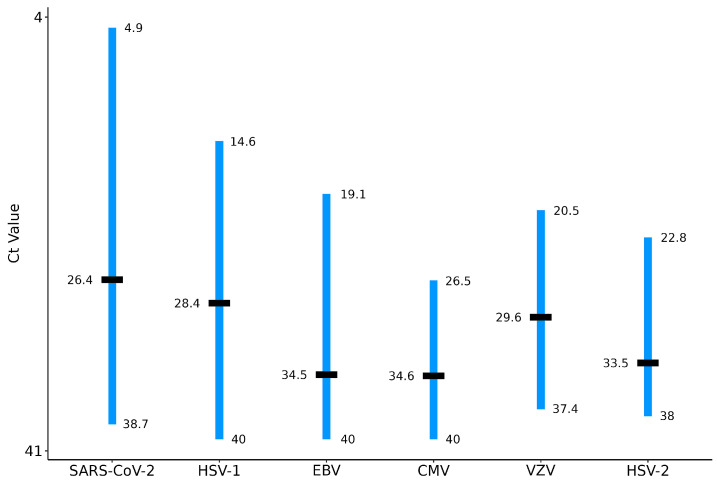
Ct-value distribution interval (blue) and median values (black lines) of positive samples for specific targets. Legend: SARS-CoV-2, severe acute respiratory syndrome coronavirus 2; HSV-1, herpes simplex virus 1; EBV, Epstein–Barr virus; CMV, cytomegalovirus; VZV, varicella zoster virus; HSV-2, herpes simplex virus 2.

Further analysis was performed to obtain better insight into the distribution of the samples according to the Ct values of the respective pathogen tested. The overall Ct value differences between the minimum and maximum Ct values were 33.8 for SARS-CoV-2, followed by 25.4 for HSV-1, 20.9 for EBV, 16.9 for VZV, 15.2 for HSV-2, 13.5 for CMV, 12.6 for *L. pneumophila*, and 4.4 for *M. pneumoniae*. The Ct value distribution intervals and median values for all pathogens tested are presented in Figure 1.

### 3.5. Combination of Co-Detections of Herpesviruses and Atypical Bacteria in SARS-CoV-2-Positive Patients

A total of 204 (89.1%) patients had at least one co-detection present. Further analysis of co-detections showed that most often, two additional pathogens were detected alongside SARS-CoV-2 in 88 (38.4%) such patients. One co-detection was observed in sixty-four (27.9%), three in fifty-one (22.3%), and four in one (0.4%) patient.

A further analysis of SARS-CoV-2-positive patients with only one additional co-detection showed that 38 (16.6%) were positive for EBV, 22 (9.6%) for HSV-1, and 4 (1.7%) for CMV. Co-detections of SARS-CoV-2 with HSV-2, VZV, *M. pneumoniae*, or *L. pneumophila* only were not observed.

Interestingly, in the group of 140 (61.1%) patients with more than one additional detection alongside SARS-CoV-2, herpesviruses were implicated in all combinations. The majority of multiple co-detections were represented by combinations of HSV-1, CMV, and EBV, which were detected in 129 (56.3%) patients. The detailed results and respective combinations are presented in Table 3.

### 3.6. Detailed Analysis of Herpesviruses Co-Detections in LRT Samples

**Figure 2:**
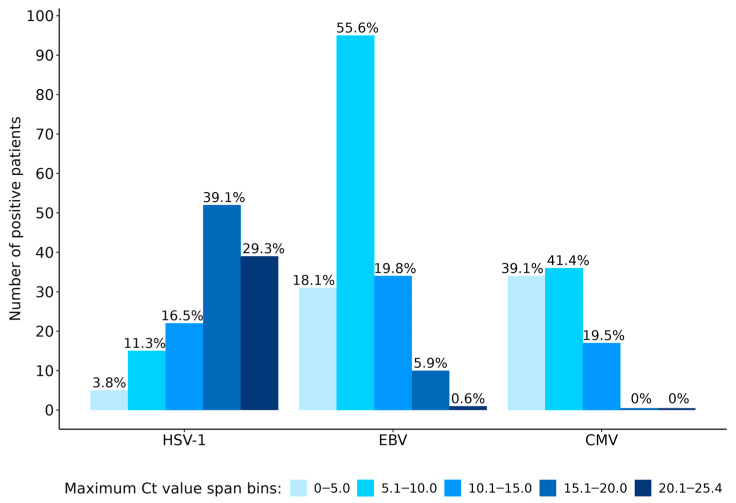
Distribution of positive patients divided into five groups for EBV, HSV-1, and CMV according to maximum Ct value spans and percentage of those patients in each group. Legend: HSV-1, herpes simplex virus 1; EBV, Epstein–Barr virus; CMV, cytomegalovirus.

Because EBV, HSV-1, and CMV were the most common co-detections in our cohort of patients, further analyses based on (rt)RT-PCR Ct values were performed for a more in-depth evaluation of these cases. The individual patients were divided into five groups based on Ct value span differences, as described in the methods section. This measure serves as a surrogate marker for putative reactivation based on the assumption that the higher the maximum Ct span, the stronger the indication of active replication/reactivation is present, rather than the change due to sample type/quality/collection. There were 96.2%, 81.9%, and 60.9% of patients with a maximum Ct value span greater than 5.0 for HSV-1, EBV, and CMV, respectively. It is interesting that the largest share of HSV-1-positive patients (39.1%) corresponded to the group with a maximum Ct value span in the range of 15.1 to 20.0, followed by 29.3% of patients in the group 20.1 to 25.4, both pointing to possible reactivation. The greater share of patients positive for EBV (55.6%) and CMV (41.4%) corresponded to the group with a Ct value span in a range of 5.1 to 10.0, which could still be indicative of presumable reactivation. On the other hand, 3.8%, 18.1%, and 39.1% of patients with HSV-1, EBV, and CMV co-detections, respectively, belonged to the Ct value span group of 0 to 5.0. Although not impossible, reactivation of respective herpesvirus for these patients is more difficult to assess. The detailed distribution of positive patients based on absolute Ct value spans is presented in Figure 2.

#### 3.6.1. Temporal Delay between SARS-CoV-2 Infection and Herpesvirus Co-Detections

**Figure 3:**
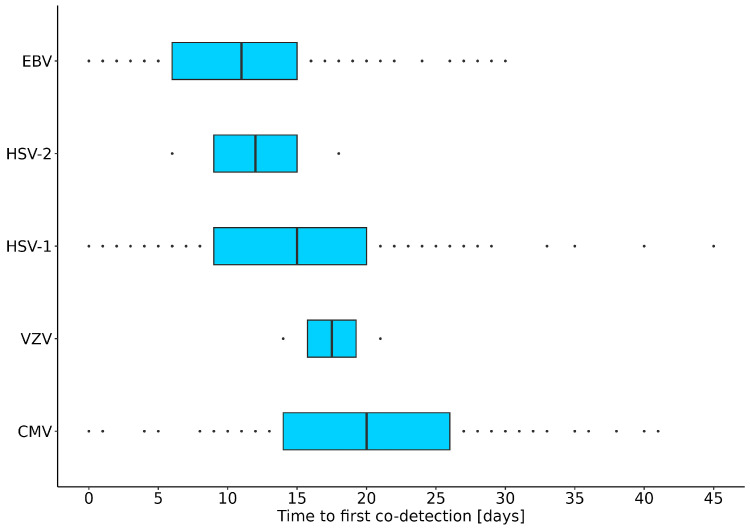
Visual presentation of time delay in days between first herpesvirus co-detection after SARS-CoV-2 initial confirmation. Vertical black lines represent median values, boxes represent the interquartile range, and dots represent the remaining quartile of results (12.5% on each side). Legend: EBV, Epstein–Barr virus; HSV-2, herpes simplex virus 2; HSV-1, herpes simplex virus 1; VZV, varicella zoster virus; CMV, cytomegalovirus.

Furthermore, for the herpesvirus family, the delay time between the 1st day of SARS-CoV-2 detection and the 1st day of herpesvirus co-detections was calculated for each target. For the 1st day of SARS-CoV-2 detection, the date of the first positive laboratory test (time point zero), from either the upper or LRT sample, was used. Our results show that, on average, it took 11 to 20 days after the first SARS-CoV-2 detection for the herpesviruses tested to be detected in LRT samples (Figure 3). In addition, for the herpesviruses detected —CMV, HSV-1, and EBV—there were 70 (80.5%), 97 (72.9%), and 86 (50.3%) positive patients, respectively, with no detection of these herpesviruses in the initial LRT samples.

#### 3.6.2. Comparison of BAL and TA Samples for Detection of HSV-1, EBV, and CMV

Because the majority of available samples for this study were TA, further analysis was performed to determine whether a detection difference for EBV, CMV, and HSV-1 could be observed in comparison to BAL samples. For this analysis, 112 patients with both sample types available were included. The time interval between the first available TA and BAL averaged 10 days, with a minimum of 0 days for patients with TA and BAL available on the same day and a maximum difference of 43 days. The detection of HSV-1 was consistent in both sample types for all 52 patients. EBV was detected in BAL of 61 (100%) and in TA of 59 (96.7%) patients. CMV was detected in BAL of 29 (100%) and in TA of 28 (96.6%) patients. Based on our cohort, no significant difference was found in the performance of HSV-1, EBV, or CMV molecular detection from BAL compared to TA.

#### 3.6.3. Clinical and Laboratory Data Correlation Analysis for Herpesvirus Co-Detection Type

The results of the maximum Ct value span comparison between patients with different clinical outcomes for HSV-1, EBV, and CMV indicate that patients that survived exhibited lower maximum Ct spans of HSV-1 and EBV. A borderline significant difference in the maximum Ct value spans for HSV-1 and EBV was observed between the survivors and the deceased. The calculated median to maximum Ct value span for HSV-1 for the deceased was 19.6 (range 3.9–25.4) and for the survivors, 17.7 (range 3.3–21), *p* = 0.09. The calculated median to maximum Ct value span for EBV for the deceased was 8.5 (range 2.7–21) and 7.4 (range 2.3–19), *p* = 0.09 for the survivors. There were no significant differences for CMV.

Next, the results of maximum Ct value span comparison between patients that received immunosuppressive treatment and those that did not for HSV-1, EBV, and CMV indicate that patients that received immunosuppressive treatment exhibited higher maximum Ct spans for HSV-1 (treated: 20.1, range 17.3–23.8; untreated: 18.4, range 3.3–25.4; *p* = 0.1). There were no significant differences for EBV and CMV.

No significant differences in median to maximum Ct value spans were calculated for HSV-1, EBV, or CMV between patients that received antiviral treatment and those that did not.

There were no significant differences in the number of co-detected herpesviruses (regardless of combination) for each paired group (survived: deceased, immunosuppressive treatment: no immunosuppressive treatment, antiviral treatment: no antiviral treatment).

## 4. Discussion

To the best of our knowledge, this study is the largest and most comprehensive longitudinal study on molecular detection of SARS-CoV-2 co-detection, with five herpesviruses and three atypical bacteria in ICU patients. Moreover, with the comparison between different sample types and times from SARS-CoV-2 confirmation to co-detections, this study offers insight into the importance of sample collection and the timeframe when to expect possible co-detection when dealing with critically ill SARS-CoV-2-positive patients. Finally, with the analysis of clinical data on disease outcome, administered immunosuppressive and antiviral treatment, and Ct value spans, this study sheds some light on whether molecular co-detection of herpesviruses in SARS-CoV-2 patients corresponds to reactivation or latent detection.

Herpesvirus reactivation in SARS-CoV-2-infected individuals with asymptomatic and mild cases of COVID-19 must be mentioned. During the SARS-CoV-2 pandemic, an increasing number of studies reported human herpesvirus reactivation [4]. The pandemic itself caused psychological stresses, psychological disorders including depression, anxiety and stress that may have contributed to herpesvirus reactivation in COVID-19 patients [4,5,6]. Additionally, the proteins or transcripts of SARS-CoV-2 can induce herpesvirus reactivation directly, by alterations in the regulation of host factors that are involved in cellular signaling pathways related to reactivation or by interactions with herpesvirus elements. In addition, SARS-CoV-2 infection can alter the immune system of the patient, causing the cytokine storm, which is especially important in severely ill COVID-19 patients that we have investigated in our study, since immunosuppression caused by SARS-CoV-2 may cause herpesvirus reactivation [4,5,6].

Studies of SARS-CoV-2-positive patients with herpesvirus coinfections have been previously performed [4,26] and showed coinfections with HSV-1 in 11.1% to 50.8% of cases [15,22,27,28,29], CMV in 15% to 41.4% [14,15,16,27,28,29], and EBV in 13.3% to 82% [15,16,19,20,21], as determined from blood samples. Substantial data are available from studies focused on coinfections of SARS-CoV-2 patients with HSV-1 and CMV detected from LRT samples by PCR. The results show a wide range of coinfections because HSV-1 and CMV were detected in 18.6% to 83.3% [4,5,13,17,18,22,23,24,25] and 7.8% to 42% [4,5,17,18] of SARS-CoV-2-positive patients, respectively. On the other hand, to the best of our knowledge, studies analyzing EBV detection in LRT samples of SARS-CoV-2 patients are scarce [4]. The results of our study, which also show a high percentage of ICU SARS-CoV-2 patients with co-detection of HSV-1 and CMV, correspond to the previously available data, which is unsurprising considering the high percentage of the population with latent herpesvirus infections [4]. For co-detections with EBV, our results show 74.7% of cases, which is higher than determined in a meta-analysis of similar studies showing a cumulative incidence of EBV among SARS-CoV-2 patients of 45% [4]. A possible reason for the higher percentage in our study is that co-detections were analyzed in multiple consecutive samples for each individual patient (Table 1) with no Ct cutoff for a positive sample, providing more analytical information, and that disease severity varied among different studies, which can also influence herpesvirus reactivations and consequently detection. Considering VZV, there are very limited data on VZV co-detections in LRT samples of SARS-CoV-2-positive patients [18,26]; mostly case reports of VZV reactivation after SARS-CoV-2 vaccination are available [4,30]. To the best of our knowledge, there were no reports of co-detections of HSV-2 in LRT samples of SARS-CoV-2-positive patients in previous studies [13,22], which is in line with our study, which showed co-detection in only one patient.

Several previous studies, also reporting concomitant herpesvirus infections regardless of detection method [4,15,16,17], are in line with our results, which showed co-detections with more than one herpesvirus in 60.7% of all patients (Table 3).

In addition, because the most common co-detections in our study were EBV, HSV-1, and CMV, we calculated that on average, it takes 11 to 20 days after the first SARS-CoV-2 detection for these herpesviruses to be detected in LRT samples (Figure 3). Generally, the most representative LRT sample for co-detection/infection and reactivation studies is BAL; however, in our retrospective study, TA, BA, and SP samples were also included because only a limited number of BAL samples were available. Due to this fact, we compared the efficacy of herpesvirus detection in BAL versus TA. Our result showed consistent detection of HSV-1, EBV, and CMV in TA compared to BAL in patients for which both types of samples were available. These findings are further supported by a previous study, which indicates that from a metagenomic perspective, TA sampling is an effective alternative to more invasive mini-BAL testing for patients with pneumonia [31].

Furthermore, Ct value distribution analysis supports the indication for possible reactivation of these herpesviruses because the Ct value difference among the patient samples resulted in several log changes for individual targets (Figure 1). Further detailed distribution of patients with co-detections of EBV, HSV-1, and CMV according to maximum Ct value spans in individual patient samples showed that 96.2%, 81.9%, and 60.9% of patients had a maximum Ct value span of more than 5.0 for HSV-1, EBV, and CMV, respectively (Figure 2). The contribution of EBV reactivation to respiratory pathology is still controversial [32,33], and HSV-1 and CMV can cause pneumonia in immunosuppressed patients [33]. Regarding HSV-1, its causative role in lung involvement is not completely understood, whether isolation from LRT samples is due to viral shedding from the upper respiratory tract or real HSV-1 bronchopneumonitis [33,34,35]. We acknowledge that during latent infection, viral DNA of EBV can be detected in B lymphocytes and epithelial cells of the oropharynx, and DNA of CMV in leukocytes, and that DNA detection alone does not necessarily represent active replication [32,33,36,37]; however, for groups of patients with maximum Ct spans for HSV-1, EBV, and CMV of more than 5.0, active replication could be suspected. In addition, our results showed a high percentage of positive patients—80.5%, 72.9%, and 50.3%—without detection of CMV, HSV-1, and EBV in the initial LRT samples, respectively, which further indicates suspected active replication/reactivation of these herpesviruses. Serology data also strongly support reactivation of these herpesviruses, since the majority of patients with HSV-1, CMV or EBV detection in LRT samples already had IgG antibodies.

Nevertheless, although the number of patients receiving antiviral therapy was rather small, all patients positive for HSV-1, and almost all those for CMV and EBV, who were selected for specific antiviral therapy belonged to groups with a higher maximum Ct span.

Considering LRT infections, we were also interested in concurrent infections with *C. pneumoniae*, *M. pneumoniae*, and *L. pneumophila*/spp. There are previous studies of *L. pneumophila/*spp. [7,18,38,39] and *M. pneumoniae* [8,10,11,18] coinfections in SARS CoV-2-positive patients for whom there was no detection of tested bacteria, or the incidence was less than 3%. In our study, *L. pneumophila* and *M. pneumoniae* were detected in LRT samples in only one patient (Table 3). The *L. pneumophila*-positive patient also had a positive urinary antigen test. Our results are in line with the findings of a meta-analysis in which *L. pneumophila* was confirmed in only 0.4% of patients by PCR in respiratory samples or by urinary antigen test [7] Coinfections of SARS CoV-2 with *C. pneumoniae* were previously described [8,9]; however, data on LRT samples are scarce. There was no detection of *C. pneumoniae* in our study. Altogether, our results concur with the previously available data, suggesting that atypical bacteria coinfections in ICU SARS-CoV-2-positive patients are rare.

Finally, taking all the laboratory data regarding HSV-1, CMV, and EBV together, no statistically significant differences could be observed regarding available clinical data. However, when interpreting these results, one should keep in mind that this was a retrospective study and that only a small subset of the patients included received immunosuppressive or antiviral therapy. There was no monitoring of viremia levels for CMV and EBV in our study and no data on comorbidities were collected.

The further limitations of our study are differences in the number of samples per patient and differences in collection times during disease progression because the decision for sampling was made by clinicians based on each patient’s clinical presentation. It is important to bear in mind that Ct values may be affected by the sample collection and its quality, since multiple physicians collected the samples. On the other hand, the strengths of our study were that we performed only one total nucleic acid isolation for each sample to minimize the impact of multiple processing of the same sample. Moreover, all the samples from an individual patient were analyzed in the same PCR run. We also included all the data in our analyses regarding Ct values to provide as much analytical information as possible.

## 5. Conclusions

In conclusion, in our cohort of severely ill COVID-19 patients treated at the ICU, HSV-1, EBV, and CMV were very frequently detected in LRT samples (in 89.1% of patients), whereas HSV-2, VZV, and atypical bacteria were found only rarely (in less than 1% of patients). Most of the co-detections observed involved two pathogens in addition to SARS-CoV-2. On average, herpesviruses were detected 11 to 20 days after the first SARS-CoV-2 confirmation. Our results showed consistent detection of HSV-1, EBV, and CMV in TA compared to BAL samples in patients for whom both sample types were available. Furthermore, our results indicate that SARS-CoV-2 infection could be one of the risk factors for latent herpesvirus reactivation in LRT of severely ill patients admitted to the ICU, especially for HSV-1, EBV, and CMV, as shown by maximum Ct value spans supported by the serology data. To further evaluate these laboratory results in a clinical context, prospective studies are needed for better determination of the true impact of herpesviruses or atypical bacteria coinfections on the prognosis and treatment of SARS-CoV-2-positive patients.

## Figures and Tables

**Table 1:** Number of patients and consecutive follow-up samples.

Patients, n	16	40	44	44	28	19	8	15	1	2	5	2	1	2	1	1
Consecutive samples per patient, n	3	4	5	6	7	8	9	10	11	12	13	14	15	16	17	18

**Table 2:** Detailed data of antiviral and immunosuppressive treatment for all patients (*N* = 229).

Treatment Type	Antiviral Agent	Patients, n (%)
Immunosuppressive treatment		22 (9.6)
Antiviral treatment	Acyclovir	21 (9.2)
	Valganciclovir/ganciclovir	12 (5.2)
	Acyclovir + ganciclovir	1 (0.4)
Immunosuppressive + antiviral treatment		12 (5.2)
		**Mean duration, days (min–max)**
Antiviral treatment	Acyclovir	11 (3–16)
	Valganciclovir/ganciclovir	25 (6–47)

**Table 3:** SARS-CoV-2-positive patients with more than one co-detection (*N* = 229).

Target 1	Target 2	Target 3	Target 4	Target 5	Patients with Co-Detections, n (%)
SARS-CoV-2	HSV-1	EBV			53 (23.1)
SARS-CoV-2	HSV-1	EBV	CMV		48 (21.0)
SARS-CoV-2	EBV	CMV			28 (12.2)
SARS-CoV-2	HSV-1	CMV			6 (2.6)
SARS-CoV-2	HSV-1	EBV	CMV	VZV	1 (0.4)
SARS-CoV-2	HSV-1	EBV	HSV-2		1 (0.4)
SARS-CoV-2	HSV-1	EBV	VZV		1 (0.4)
SARS-CoV-2	HSV-1	Mpn			1 (0.4)
SARS-CoV-2	HSV-2	EBV	Lpn		1 (0.4)

Legend: SARS-CoV-2, severe acute respiratory syndrome coronavirus 2; HSV-1, herpes simplex virus 1; EBV, Epstein–Barr virus; HSV-2, herpes simplex virus 2; CMV, cytomegalovirus; Mpn, *Mycoplasma pneumoniae*; VZV, varicella zoster virus; Lpn, *Legionella pneumophila*.

## Data Availability

The datasets used and analyzed during the current study are available from the corresponding author on reasonable request.

## Appendix A. Detailed Description of Methods

### Appendix A.1. Total Nucleic Acid Isolation

Total nucleic acids (NA) were isolated from 200 μL of each sample using the MagNA Pure 96 DNA and Viral NA Small Volume Kit (Roche Applied Science, Mannheim, Germany) according to the manufacturer’s instructions in an automated MagNA Pure 96 (Roche Applied Science, Mannheim, Germany) instrument, with the elution volume set to 50 μL. The purified NA were used immediately or stored at −20 °C until further testing.

### Appendix A.2. (rt)RT-PCR

A Janus G3 pipetting robot (Perkin Elmer, Waltham, MA, USA) was used for automatic dispensing of each master mix and samples for an individual run of rtRT-PCR or RT-PCR as suitable per pathogen.

Reverse transcription, amplification, and detection for SARS-CoV-2 were performed in a QuantStudio™ 5 System (Applied Biosystems, Waltham, MA, USA). A LightCycler 480 instrument (Roche Applied Science, Mannheim, Germany) was used for RT-PCR for detection of members of the *Herpesviridae* family and atypical bacteria detection.

### Appendix A.3. SARS-CoV-2 rtRT-PCR

For detection of SARS-CoV-2, multiplex rtRT-PCR was performed using a Lightmix^®^ Kit SARS-CoV-2 E+N UBC kit (Roche Applied Science, Mannheim, Germany). The master mix was composed of 2.5 µL 1 × TaqMan^®^FastVirus 1-Step Master Mix (Thermo Fisher Scientific, Waltham, MA, USA), 0.25 µL Lightmix^®^ Kit SARS-CoV-2 E+N UBC (Roche Applied Science, Mannheim, Germany), 4.75 µL ddH2O (Promega, Madison, WI, USA), and 5.0 µL total NA. Cycling conditions for rtRT-PCR were set as follows: 5 min at 50 °C, 20 s at 95 °C, and 45 cycles of 3 s at 95 °C and 30 s at 60 °C.

### Appendix A.4. VZV, EBV, CMV, HSV-1, and HSV-2 RT-PCR

Commercially available RT-PCR kits (GeneProof a. s., Brno, Czech Republic) were used to perform RT-PCR for selected members of the *Herpesviridae* family. The GeneProof Varicella-Zoster Virus (VZV) PCR Kit, GeneProof Epstein–Barr Virus (EBV) PCR Kit, and GeneProof Cytomegalovirus (CMV) PCR Kit were used for singleplex detection of VZV, EBV, and CMV, respectively. The Multiplex GeneProof Herpes Simplex Virus (HSV-1/2) PCR Kit was used for the simultaneous detection and differentiation of HSV-1 and HSV-2. Each reaction contained 7.5 µL of ready-to-use master mix and 2.5 µL of total nucleic acids. RT-PCR cycling conditions were set as follows: 2 min at 37 °C, 10 min at 95 °C, and 45 cycles of 5 s at 95 °C, 40 s at 60 °C, and 20 s at 72 °C.

### Appendix A.5. Chlamydia pneumoniae, Mycoplasma pneumoniae, and Legionella pneumophila/spp. RT-PCR

Qualitative multiplex RT-PCR was performed to detect *Chlamydia pneumoniae*, *Mycoplasma pneumoniae*, and *Legionella pneumophila/*spp. Each amplification reaction contained 2.0 µL LightCycler^®^ Multiplex DNA Master (Roche Applied Science, Mannheim, Germany), 0.25 µL LightMix^®^ Modular *Chlamydia pneumoniae* (TIB Molbiol, Berlin, Germany), 0.25 µL LightMix^®^ Modular *Mycoplasma pneumoniae* (TIB Molbiol, Berlin, Germany), 0.25 µL LightMix^®^ Modular *Legionella* spp./pn. (TIB Molbiol, Berlin, Germany), 2.25 µL ddH_2_O (Promega, Madison, WI, USA), and 5.0 µL total NA. The cycling conditions were as follows: 5 min at 95 °C, 45 cycles of 5 s at 95 °C, 15 s at 60 °C, and 15 s at 72 °C, and 30 s at 40 °C. Melting curve analysis was performed to distinguish between *Legionella* spp. and *Legionella pneumophila*. The temperature was first held at 95 °C for 30 s and then gradually increased from 40 °C to 85 °C at a ramp rate of 1.5 °C/s.

## Appendix B

### Clinical Criteria for Evaluation of Severe Disorder of the Immune System

Clinical criteria that were used for determination of patients with severe disorder of the immune system are presented in Table A1.

**Table A1:** Clinical criteria for determination of patients with severe disorder of the immune system.

* Cause of ID: Acquired/Iatrogenic/Drug-Induced	Congenital ID
HPSCT (<12 months)	
GvHD	XLA
HIV-infection < 200 CD4/mm^3^	IFN
Induction chemotherapy in pediatric leukemia	IgE sy
Chemotherapy	CVID
Solid organ transplantation	CGD
Immunosuppressive medications *	Wiskott–Aldrich syndrome

Legend: ID, immune disorder; HPSCT, hematopoietic stem cell transplantation; GvHD, graft-versus-host disease; XLA, X-linked agammaglobulinemia; IFN, interferon receptor deficiency; IgE sy, hyper IgE syndrome; CVID, common variable immunodeficiency; CGD, chronic granulomatous disease. * rituximab and other biologics that act against B lymphocytes (anti-CD20, anti-CD38, anti-CD52, proteasome inhibitors, chimeric antigen receptor (CAR) T-cell cell therapy directed against antigens on the surface of B lymphocytes); antimetabolites, alkylating agents, methylprednisolone (or equivalent) ≥ 16 mg > 14 days.
